# Dexmedetomidine versus propofol sedation in flexible bronchoscopy: a randomized controlled trial

**DOI:** 10.1186/s12890-022-01880-9

**Published:** 2022-03-15

**Authors:** Barak Pertzov, Boris Krasulya, Karam Azem, Yael Shostak, Shimon Izhakian, Dror Rosengarten, Svetlana kharchenko, Mordechai R. Kramer

**Affiliations:** 1 Pulmonary Division, Rabin Medical Center, Beilinson Campus, 49100 Petach-Tikvah, Israel; 2 Department of Anesthesia, Rabin Medical Center, Petach Tikva, Israel; 3 Department of Anesthesia, Hasharon Hospital, Rabin Medical Center, Petach Tikva, Israel; 4 Sackler Faculty of Medicine, Tel Aviv University, Tel Aviv, Israel

**Keywords:** Dexmedetomidine, Flexible bronchoscopy, Sedation, Desaturation, Adverse events

## Abstract

**Background:**

Dexmedetomidine (DEX), is a highly selective alpha2 adrenoceptor (α2-AR) agonist, successfully used in various procedures including flexible bronchoscopy. Randomized controlled trials (RCTs) evaluating DEX sedation during bronchoscopy report equivocal results regarding respiratory and hemodynamic outcomes.

**Methods:**

We conducted an RCT to evaluate the efficacy and safety of dexmedetomidine compared to propofol for sedation during bronchoscopy. The primary outcome was the number of desaturation events, secondary outcomes were transcutaneous Pco2 level, hemodynamic adverse events and physician and patient satisfaction.

**Results:**

Overall, 63 patients were included, 30 and 33 in the DEX and propofol groups, respectively. The number of desaturation events was similar between groups, median (IQR) 1 (0–1) and 1 (0–2) in the DEX and control groups, respectively (P = 0.29). Median desaturation time was 1 (0–2) and 1 (0–3) minutes in the DEX and control groups, respectively (P = 0.48). Adverse events included hypotension, 33% vs 21.1% in intervention and control groups, respectively (P = 0.04), bradycardia, cough, and delayed recovery from sedation. Total adverse events were 22 and 7 in DEX and propofol groups, respectively (P = 0.009).

**Conclusion:**

Dexmedetomidine sedation during bronchoscopy did not show differences in oxygen saturation and transcutaneous CO2 level in comparison to propofol. Moreover, DEX sedation required a significantly higher number of rescue boluses, due to inadequate sedation and was associated with a higher rate of adverse events.

*Trial registration* NCT04211298, registration date: 26.12.2019.

## Background

Fiberoptic bronchoscopy (FOB) is commonly used for the diagnosis and management of a variety of lung diseases [1, 2]. The procedure is usually performed under sedation, with a selection of available methods to provide different depths and lengths of sedation, according to the type of procedure and operator preference [3, 4]. Dexmedetomidine (DEX) is a highly selective alpha2 adrenoceptor (α2-AR) agonist, it has sedative, anxiolytic and analgesic qualities. Following intravenous administration, DEX has a distribution half-life of six minutes and a terminal elimination half-life (t½) of approximately two hours. Reported adverse events include both hypotension and hypertension, bradycardia, dry mouth and nausea [5, 6]. DEX has been successfully used in a wide array of procedures including colonoscopy, endoscopic retrograde cholangio-pancreatography (ERCP), laparoscopic procedures, shockwave lithotripsy, awake carotid endarterectomy, retinal surgery and in pediatric patients [7–15]. In 2012 Ryu et al. conducted the first randomized controlled trial (RCT) that evaluated the use of DEX during bronchoscopy. The DEX group had a significantly lower rate of desaturation events, with no between group difference in level of sedation, oxygen saturation, mean arterial pressure and heart rate [16]. During the period from 2012 to 2021 eleven more RCTs were conducted to evaluate DEX during bronchoscopy, in comparison to propofol, midazolam and fentanyl, in various combinations. While some of these trials reported a lower rate of desaturation events and an adequate sedation level, others reported no difference in desaturation events, a higher rate of hemodynamic adverse events and an inferior level of patient sedation and bronchoscopist satisfaction [17–27]. Therefore, DEX sedation for bronchoscopy is considered an emerging method for sedation during bronchoscopy. However, whether DEX sedation conveys a lower rate of desaturation events or adequate sedation level is yet to be determined. In this study we conducted an RCT to evaluate the efficacy and safety of dexmedetomidine sedation in comparison to propofol sedation during bronchoscopy.

## Methods

We conducted a single center, RCT, that compared the use of dexmedetomidine to propofol, as the main drug for sedation during bronchoscopy. Patients were recruited between December 2019 to April 2020 and from January to May 2021. During these time intervals adult patients (age > 18 years) who were scheduled to undergo Bronchoscopy at Rabin Medical Center (RMC) in Israel, were offered to participate in the study. Exclusion criteria included: known or suspected allergy to any of the study drugs, seizure disorder, renal impairment (with serum creatinine > 2 mg/dL) or hepatic impairment (elevated liver enzymes > 2 times normal levels), hemodynamic instability (bradycardia with HR < 50 bpm or hypotension with SBP < 90 mmHg), or seriously ill patients with American Society of Anesthesiologists’ (ASA) physical status above III. The study was approved by RMC institutional review board (IRB) (RMC-0312-19) and registered in a clinical trial registry (NCT04211298, registration date: 26.12.2019). All methods were carried out in accordance with the CONSORT guidelines and regulations. After signing an informed consent form, the patient was randomized with computer generated random numbers, sealed in opaque envelopes, to either DEX group or propofol group.

### Sedation protocol

An anesthesiologist was present throughout each procedure and oversaw monitoring and sedation protocol in all cases, a pulmonologist and a nurse were also present in all procedures. The number of procedures each day was between 3 to 4. Monitoring included continuous electrocardiography, pulse oximetry, transcutaneous PCO2 and automated noninvasive blood pressure recordings. All patients received supplemental nasal oxygen at 2–5 l/min. The port of entry was usually nasal, laryngeal mask airway (LMA) was used in endobronchial ultrasonography (EBUS) procedures. Rigid bronchoscopy was not used in this study. The sedation protocols for both groups included a loading dose of fentanyl 1 mcg/kg and midazolam 1 mg. Patients randomized to the DEX group received a loading dose of 1 mcg/kg over 15 min followed by a continuous intravenous infusion at a rate of 0.5 mcg/kg/h. Patients in the propofol group received a dose of 0.5–1 mg/kg for induction over 1 min followed by a maintenance infusion in a dose of 100–200 mcg/kg/min. In both groups bolus doses of propofol of 0.1–0.5 mg/kg were given for insufficient sedation. Topical anesthesia on the vocal cord and carina, with 1% lidocaine, was used in all patients.

### Outcomes

The primary outcomes were the number of de-saturation events, during bronchoscopy and the time in which the oxygen saturation level decreased under 90%. Secondary outcomes were the Richmond Agitation Sedation Scale (RASS), level of transcutaneous PCO2 (PcCO2), blood pressure, number of propofol boluses given for insufficient sedation, length of procedure and adverse events. Bronchoscopist satisfaction level and patient discomfort were also evaluated on a scale of 1 to 5. For physician satisfaction 5 represents high satisfaction and 1 poor satisfaction. For patient discomfort 5 represents no discomfort and 1 maximal discomfort.

### Statistical analysis

The baseline characteristics and secondary outcomes were analyzed with the student’s t-test, chi-square test and the Mann–Whitney U test, as appropriate. The primary outcome was analyzed with the chi-square test for the number of desaturation events and with the Mann–Whitney U test for the time in which desaturation was recorded. A P-value of 0.05 was considered as significant. Statistical analysis was conducted with the SPSS version 27 software. Sample size was calculated with WINPEPI software, the primary outcome evaluated was the number of desaturation events per patient. Assuming a variance of 1.5 events and to detect a mean difference of 1 event with an alpha of 0.05 and a power of 80%, a sample size of 48 patients (24 in each group) is required.

## Results

Overall, 63 patients were included in the current study, 30 patients in the DEX group and 33 in the propofol group. Mean age in the intervention and control groups was 58.76 ± 15.09 and 62.96 ± 9.69, respectively (P = 0.19). Weight, ASA score and baseline CO2 were similar between groups. Male sex was more common in the propofol group, 69% vs 43% in the control and intervention groups, respectively (P = 0.03). Three patients in the propofol group were chronically treated with neuro-psychiatric medication in comparison to zero patients in the DEX group (P = 0.16). There was no difference in the distribution of COPD, IHD, CHF between treatment groups. Procedure types were balanced between groups (Table 1). The number of desaturation events was similar between groups, median (IQR) 1 (0–1) and 1 (0–2) in the intervention and control groups, respectively (P = 0.29). The median desaturation time was 1 (0–2) and 1 (0–3) minutes in the intervention and control groups respectively (P = 0.48). The median rise in PcCO2 was 18.45 (14.70–22.97) and 20.65 (15.37–30.07) mm/Hg and the median time in which PcCO2 was above 50 mm/Hg was 17.5 (14.12–27.12) and 19.5 (7.75–29.75) minutes in the intervention and control groups, respectively (P = 0.46 and P = 0.94). The median RASS score was − 2 [(− 3) to (− 2)] and − 3 [(− 3) to (− 2)] for the DEX and propofol groups, respectively (P = 0.01). Patients in the DEX group required a median of 2.5 (2–5) propofol rescue boluses during the procedure in comparison to 2 (1.0–2.5) boluses in the propofol group (P = 0.01), the median of total propofol dose of additional rescue boluses was 90 mg (50–109) and 58 mg (42–106.5) in the DEX and propofol groups, respectively (P = 0.18). Median procedure time was 20 (8–35) and 21 (15–27.5) minutes in the intervention and control groups, respectively (P = 0.70). Adverse events included hypotension, 33% vs 21.1% in the intervention and control groups, respectively (P = 0.04), post-procedural hypotension, bradycardia, cough and delayed recovery from sedation. The total number of adverse events was 22 in the DEX group and 7 in the propofol group (P = 0.009) (Table 2). The median score of physician satisfaction from sedation during procedure was 4.5 (4–5) and 5 (5–5) and the median score of patient discomfort was 5 (4.5–5) and 5 (5–5) in the intervention and control groups, respectively (P = 0.01 and P = 0.1).

**Table 1:** Demographic and baseline clinical characteristics

	Dexmedetomidine	Propofol	P value
n	30	33	
Age*	58.76 ± 15.09	62.96 ± 9.69	0.19
Male gender	13 (43.3)	23 (69.7)	0.03
Weight*	76.43 ± 18.99	75.73 ± 15.44	0.87
ASA score*	2 (2–2)	2 (2–3)	0.47
Baseline CO2*	35.27 ± 9.20	37.40 ± 8.66	0.41
COPD	6	8	0.86
IHD	1	3	0.38
CHF	0	2 (6.3)	0.30
neuro-psychiatric medication^#^	3	0	0.16
Procedure type			
BAL	9	5	0.55
Bronchoscopy + TBB	7	9	
Bronchoscopy + Cryo TBB	6	5	
EBUS	5	9	
Laser/balloon dilation	3	5	

ASA – American Society of Anesthesiologists; BAL – bronchoalveolar lavage; TBB – transbronchial biopsy; EBUS – endobronchial ultrasound; COPD – chronic obstructive pulmonary disease; IHD – ischemic heart disease; CHF – chronic heart failure

*Data presented in mean ± SD or median + IQR

^#^Antiepileptic drugs, Psychotropic Drugs

**Table 2:** Clinical outcomes for the intervention (dexmedetomidine) and control (propofol) groups

	Dexmedetomidine N = 30	Propofol N = 33	P value
Number of desaturation events	1 (0–1)	1 (0–2)	0.29
Total desaturation time	1 (0–2)	1 (0–3)	0.48
Number of patients with any desaturation events (%)	16 (53)	21(63)	0.4
PcCO2 rise during procedure, mm/Hg	18.45 (14.70–22.97)	20.65 (15.37–30.07)	0.46
Time period of PcCO2 > 50 mm/Hg in minutes	17.5 (14.12–27.12)	19.5 (7.75–29.75)	0.94
Maximal level of PcCO2 during procedure mm/Hg	56.45 (48.85–61.42)	55.20 (52.82–68.37)	0.53
RASS	− 2 [(− 3) to (− 2)]	− 3 [(− 3) to (− 2)]	0.01
Procedure time	20 (8–35)	21 (15–27.5)	0.7
Number of Propofol boluses needed	2.5 (2–5)	2 (1.0–2.5	0.016
Total bolus dose	90 (50–109)	58 (42–106.5)	0.18
Patient satisfaction ^#^	5 (4.5–5)	5 (5–5)	0.10
Bronchoscopist satisfaction ^#^	4.5 (4–5)	5 (5–5)	0.012
Adverse events (%)			
Hypotension during procedure	10 (33.3)	4 (12.1)	0.04
Bradycardia	2 (6.7)	0	0.22
Cough	2 (6.7)	0	0.22
Post-procedural Hypotension	5 (16.7)	3 (9.1)	0.3
Delayed recovery from sedation	3 (10)	0	0.10
Total*	22	7	0.009

Data presented as median (IQR)

RASS – Richmond Agitation-Sedation Scale; Pc﻿CO2: Transcutaneous CO2 partial pressure

*Five patients had 2 adverse events

^#^ 1- low satisfaction, 5- high satisfaction

## Discussion

In this study we have evaluated dexmedetomidine sedation during bronchoscopy and whether its mechanism of action, which does not involve central respiratory drive depression, has a favorable effect on the respiratory and hemodynamic adverse events. The results showed, that in comparison to propofol sedation, there was no difference in oxygen saturation, both in the number of desaturation events and in the total desaturation time (in which the oxygen saturation was lower than 90%). The level of CO2 was also similar between treatment groups, there was no difference in the median rise in PcCO2 during procedure and in the total time period in which the PcCO2 level was above 50 mm/Hg. The DEX group however, showed inferior performance in the adequacy of sedation, with a significantly higher RASS score and a significantly higher number of propofol rescue boluses during the procedure. Moreover, use of DEX was associated with a significantly higher frequency of hypotension events and total adverse events. Finally, the level of physician satisfaction was significantly lower in the DEX group.

A review of the literature shows 12 published RCT’s that evaluated DEX sedation for bronchoscopy [16–27]. Four trials evaluated DEX for conscious sedation and administered the drug only once at the beginning of the procedure [17, 23, 25, 26]. The largest of these trials by Zhang et al. reported a lower rate of desaturation events in the DEX group however, with a higher rate of adverse events, correspondingly to the current trial. One trial evaluated DEX for general anesthesia and the other 7 trials evaluated DEX for moderate/deep sedation, of these, five reported no difference in the rate of desaturation events, correspondingly to the current trial. While two trials reported a significantly lower rate of desaturation events with DEX [16, 20] (Table 3). The reported rate of adverse events varies between trials, some report a higher rate with DEX, as seen in the current trial, while some report lower rates. These inconsistences between trials can be explained by different dosing, additional drugs used for sedation, different comparators, and types of procedures. In the current trial we used midazolam and fentanyl for induction and propofol rescue boluses in both arms. The use of additional drugs can introduce potential confounding factors, (e.g., paradoxical agitation with benzodiazepine and respiratory drive depression, due to fentanyl and propofol use). Nonetheless, these confounding factors would not likely significantly impact the results regarding respiratory outcomes or have increased the rate of adverse events. Additional outcomes evaluated in the current study, included the level of sedation obtained and bronchoscopist satisfaction. For these we found DEX was inferior to propofol. Conversely, most other similar trials reported no difference in the level of sedation, apart from one trial that reported superior sedation with DEX in comparison to midazolam [19–22].

**Table 3:** Literature review—randomized controlled trials who evaluated dexmedetomidine sedation in flexible bronchoscopy procedures

References	Design	Procedure	Intervention (n)	Comparator (n)	DEX dose	Primary outcome
Ryu et al. [16]	RCT	FB	PRF– DEX (36)	PRF –rFEN (36)	0.4–2 μg/kg /H	SpO2
Liao et al. [27]	RCT	FB	DEX (99)	MDZ (99)	0.5 μg/kg /H	SpO2
Goneppanavar 2015^26^	RCT	FB	DEX (27)	MDZ (27)	1 μg/kg once	Other^a^
Yuan et al. [18]	RCT	FB	DEX-FEN (50)	PRF-FEN (50)	0.2–0.7 μg/kg /H	SpO2
Riachy et al. [25]	RCT	FB	LID + DEX (53)	LID (54) ID + FEN (55)	0.5 μg/kg once	Other ^b^
Li et al. [24]	RCT	FB	DEX + FEN + PRF (57)	FEN + PRF (57)	0.5–1 mg/kg once	SpO2
St-Pierre et al. [20]	RCT	EBUS-TBNA	DEX (30)	rFEN (30)	0.5–1.0 μg/kg /H	SpO2
Magazine et al. [23]	RCT	FB	DEX (27)	MDZ (27)	0.5 μg/kg once	Other ^a^
Lin et al. [19]	RCT	EBUS-TBNA	DEX (25)	PRF (25)	0.5–1.4 μg/kg /H	SpO2
Kim et al. [22]	RCT	EBUS-TBNA	DEX (48)	MDZ (54)	0.25–0.75 μg/kg /H	SpO2
Kumari et al. [21]	RCT	EBUS-TBNA	DEX (99)	MDZ (98)	0.6 μg/kg /H	Other ^c^
Zhang et al. [17]	RCT	FB	DEX (222)	FEN (211)	1 μg/kg once	SpO2
Pertzov 2021 (current study)	RCT	FB	DEX (30)	PRF (33)	0.5 μg kg /H	SpO2

FB – flexible bronchoscopy; CS – Conscious sedation; GA – general anesthesia; MDS – Moderate to deep sedation; DEX – dexmedetomidine; PRF – propofol; MDZ – midazolam; FEN – fentanyl; rFEN – remifentanil; LID – lidocaine; EBUS-TBNA– endobronchial ultrasound ‐guided transbronchial needle aspiration

^a^Composite outcome: sedation, cough, calmness, respiratory response, physical movement, facial tension

^b^Procedure tolerance, sedation level, safety

^c^Number of rescue midazolam boluses

The current combined evidence cannot afford us to ascertain whether DEX sedation is superior to other sedation regimens used during bronchoscopy. Nonetheless, this trial contributes to the growing body of evidence, together with a group of small RCTs, albeit demonstrating inconclusive results regarding DEX sedation during bronchoscopy. Additional large RCT’s are warranted to characterize the groups of patients and types of procedures that will confer the most benefit from DEX sedation. Moreover, since Dexmedetomidine is intended to assist in patients with high respiratory risk, it should also be evaluated with noninvasive respiratory assistance, such as high flow nasal cannula and CPAP/BiPAP devices [28, 29].

The main limitations of this study are its small sample size, lack of blinding, the use of additional drugs for sedation and inclusion of heterogenous types of procedures. However, all procedures were done with moderate sedation and although additional drugs for sedation were used, the sedation regimen and dosing were identical in all patients.

In conclusion, DEX sedation during bronchoscopy did not show differences in oxygen saturation and transcutaneous CO2 level in comparison to propofol. Moreover, DEX sedation required a significantly higher number of rescue boluses, due to inadequate sedation and was associated with a higher rate of adverse events.

## Data Availability

The datasets used and/or analyzed during the current study available from the corresponding author on reasonable request.

## Abbreviations

DEX: Dexmedetomidine
RCT: Randomized controlled trials
IQR: Inter quartile range
CI: Confidence interval
FOB: Fiberoptic bronchoscopy
ERCP: Endoscopic retrograde cholangio-pancreatography
RMC: Rabin medical center
IRB: Institutional review board
PcCO2: Transcutaneous carbon dioxide partial pressure
COPD: Chronic obstructive pulmonary disease
IHD: Ischemic heart disease
CHF: Chronic heart failure
CPAP: Continuous positive airway pressure
BiPAP: Bilevel positive airway pressure
